# Dietary patterns are related to cognitive functioning in elderly enriched with individuals at increased risk for Alzheimer’s disease

**DOI:** 10.1007/s00394-020-02257-6

**Published:** 2020-05-29

**Authors:** L. M. P. Wesselman, D. Melo van Lent, A. Schröder, O. van de Rest, O. Peters, F. Menne, M. Fuentes, J. Priller, E. J. Spruth, S. Altenstein, A. Schneider, K. Fließbach, S. Roeske, S. Wolfsgruber, L. Kleineidam, A. Spottke, V. Pross, J. Wiltfang, R. Vukovich, A. K. Schild, E. Düzel, C. D. Metzger, W. Glanz, K. Buerger, D. Janowitz, R. Perneczky, M. Tatò, S. Teipel, I. Kilimann, C. Laske, M. Buchmann, A. Ramirez, S. A. M. Sikkes, F. Jessen, W. M. van der Flier, M. Wagner

**Affiliations:** 1 German Center for Neurodegenerative Disorders (DZNE), Bonn, Venusberg-Campus 1, 53127 Bonn, Germany; 2 Department of Neurology, Alzheimer Center Amsterdam, Amsterdam Neuroscience, Vrije Universiteit Amsterdam, Amsterdam UMC, Amsterdam, The Netherlands; 3 The Glenn Biggs Institute for Alzheimer’s and Neurodegenerative Diseases, UT Health, San Antonio, TX USA; 4 Department of Neurology, Boston University, Boston, MA USA; 5 The Framingham Heart Study, Framingham, MA USA; 6 Division of Human Nutrition and Health, Wageningen University and Research, Wageningen, The Netherlands; 7 German Center for Neurodegenerative Diseases (DZNE), Berlin, Germany; 8 Charité-Universitätsmedizin Berlin, corporate member of Freie Universität Berlin, Humboldt-Universität zu Berlin, and Berlin Institute of Health, Institute of Psychiatry and Psychotherapy, Hindenburgdamm 30, 12203 Berlin, Germany; 9 Department of Psychiatry and Psychotherapy, Charité, Charitéplatz 1, 10117 Berlin, Germany; 10 Department for Neurodegenerative Diseases and Geriatric Psychiatry, University Hospital Bonn, Venusberg-Campus 1, 53127 Bonn, Germany; 11 Department of Neurology, University of Bonn, Venusberg-Campus 1, 53127 Bonn, Germany; 12 Study Center Bonn, Medical Faculty, Venusberg-Campus 1, 53127 Bonn, Germany; 13 German Center for Neurodegenerative Diseases (DZNE), Goettingen, Germany; 14 Department of Psychiatry and Psychotherapy, University Medical Center Goettingen, University of Goettingen, Von-Siebold-Str. 5, 37075 Goettingen , Germany; 15 Department of Psychiatry, University of Cologne, Medical Faculty, Kerpener Strasse 62, 50924 Cologne, Germany; 16 German Center for Neurodegenerative Diseases (DZNE), Magdeburg, Germany; 17 Institute of Cognitive Neurology and Dementia Research (IKND), Otto-Von-Guericke University, Magdeburg, Germany; 18 Department of Psychiatry and Psychotherapy, Otto-Von-Guericke University, Magdeburg, Germany; 19 German Center for Neurodegenerative Diseases (DZNE, Munich), Feodor-Lynen-Strasse 17, 81377 Munich, Germany; 20 Institute for Stroke and Dementia Research (ISD), University Hospital, LMU Munich, Feodor-Lynen-Strasse 17, 81377 Munich, Germany; 21 Department of Psychiatry and Psychotherapy, University Hospital, LMU Munich, Munich, Germany; 22 Munich Cluster for Systems Neurology (SyNergy) Munich, Munich, Germany; 23 Ageing Epidemiology Research Unit (AGE), School of Public Health, Imperial College London, London, UK; 24 German Center for Neurodegenerative Diseases (DZNE), Rostock, Germany; 25 Department of Psychosomatic Medicine, Rostock University Medical Center, Gehlsheimer Str. 20, 18147 Rostock, Germany; 26 German Center for Neurodegenerative Diseases (DZNE), Tübingen, Germany; 27 Section for Dementia Research, Hertie Institute for Clinical Brain Research and Department of Psychiatry and Psychotherapy, University of Tübingen, Tübingen, Germany; 28 Clinical Developmental Psychology and Clinical Neuropsychology, Faculty of Behavioural and Movement Sciences (FGB), Vrije University Amsterdam, Amsterdam, The Netherlands; 29 Department of Epidemiology and Biostatistics, Amsterdam Neuroscience, Vrije Universiteit Amsterdam, Amsterdam UMC, Amsterdam, The Netherlands

**Keywords:** Cognition, Dementia, Mediterranean diet, MIND diet, Dietary patterns

## Abstract

**Purpose:**

To investigate cross-sectional associations between dietary patterns and cognitive functioning in elderly free of dementia.

**Methods:**

Data of 389 participants from the German DELCODE study (52% female, 69 ± 6 years, mean Mini Mental State Score 29 ± 1) were included. The sample was enriched with elderly at increased risk for Alzheimer’s disease (AD) by including participants with subjective cognitive decline, mild cognitive impairment (MCI) and siblings of AD patients. Mediterranean and MIND diets were derived from 148 Food Frequency Questionnaire items, and data-driven patterns by principal component analysis (PCA) of 39 food groups. Associations between dietary patterns and five cognitive domain scores were analyzed with linear regression analyses adjusted for demographics (model 1), and additionally for energy intake, BMI, other lifestyle variables and APOe4-status (model 2). For PCA-derived dietary components, final model 3 included all other dietary components.

**Results:**

In fully adjusted models, adherence to Mediterranean and MIND diet was associated with better memory. The ‘alcoholic beverages’ PCA component was positively associated with most cognitive domains. Exclusion of MCI subjects (*n* = 60) revealed that Mediterranean and MIND diet were also related to language functions; associations with the alcoholic beverages component were attenuated, but most remained significant.

**Conclusion:**

In line with data from elderly population samples, Mediterranean and MIND diet and some data-derived dietary patterns were related to memory and language function. Longitudinal data are needed to draw conclusions on the putative effect of nutrition on the rate of cognitive decline, and on the potential of dietary interventions in groups at increased risk for AD.

**Electronic supplementary material:**

The online version of this article (10.1007/s00394-020-02257-6) contains supplementary material, which is available to authorized users.

## Introduction

The number of people living with dementia is increasing worldwide due to the aging of the population [1]. With a total of 46 million people living with dementia worldwide in 2015, this number is estimated to increase to 131.5 million by 2050 [2]. As no effective treatment for dementia exists, research on modifiable risk factors is critically important to derive health recommendations and preventive interventions. A healthy lifestyle has been suggested to be one of the modifiable risk factors for cognitive decline and dementia, and prevention studies, therefore, increasingly focus on lifestyle and cognition [3].

Nutrition is one of the lifestyle factors that could prevent or delay cognitive decline and dementia [4, 5]. It has been suggested that specific foods (e.g., e.g., fatty fish [6–10], red wine [6–8]) are positively associated with cognition, but this is not consistently reported [11]. Moreover, foods are usually not consumed in isolation. A dietary pattern comprises the intake of multiple foods and nutrients in combination. Also, investigating dietary patterns may be more informative for public health purposes, such as the development of food-based dietary guidelines. It has, therefore, been suggested that relationships between food intake and cognition should be evaluated by taking into account dietary patterns, rather than the intake of single food or nutrients [12].

Several dietary patterns have been established, such as the Mediterranean diet and Mediterranean-DASH Intervention for Neurodegenerative Delay (MIND) diet [13]. The Mediterranean diet comprises a high intake of whole grains, fruits, vegetables, fish and oils, while limiting the intake of fat, salt, alcohol, dairy and meat [14]. Adherence to the Mediterranean diet has been associated with a lower risk for cognitive decline, dementia, and AD [13]. The MIND diet is a modified hybrid of the Dietary Approach to Systolic Hypertension diet (DASH, aiming to reduce blood pressure) and Mediterranean diet, reflecting the most compelling scientific evidence on foods and nutrients that protect the brain [15, 16]. Compared to the Mediterranean diet, the MIND diet was defined to encourage healthy brain aging, e.g., e.g., promoting the intake of berries and green leafy vegetables, while limiting the intake of animal-based and highly saturated fat foods. Adherence to the MIND diet was found to be associated with lower incidence of AD [16], cognitive decline [13, 15, 17, 18] and subjective memory complaints [19].

The relationships between greater adherence to these diets and better cognition, slower cognitive decline and decreased risk for dementia could not always be replicated (e.g., e.g., [20–22]). Methodological challenges such as the sensitivity of outcome measures, the most sensitive time periods in life course and the true adherence of cultural diverse populations to the predefined diets might have contributed to these inconsistent findings [23]. A complementary strategy for the analysis of dietary patterns is a bottom-up approach, using data-driven methodologies to identify underlying dietary patterns of the specific population under study [24]. For example, a Japanese study found that a dietary pattern characterized by high intake of soybean products, vegetables and dairy products and a low intake of rice was associated with reduced risk for dementia [25]. In a Swedish sample, the ‘prudent’ pattern, characterized by vegetables, fruit, oil and fish, was associated with less decline in MMSE [26] and in an American sample, a pattern characterized by higher intakes of amongst others salad dressing, several vegetables, fruit, nuts, fish and poultry, and a lower intake of high-fat dairy products, red meat, organ meat, and butter was associated with lower risk for AD [27].

Most studies on cognition and diet have been conducted in general population-based samples and could lead to primary prevention recommendations. However, studies in individuals at increased risk for AD, e.g., e.g., subjects who report or show symptoms of cognitive decline [28, 29] may be particularly informative. These samples are expected to include more subjects with AD pathology, which could be counteracted by certain nutrients or diets, to be leveraged for secondary prevention. A recent study in memory-clinic patients with subjective cognitive decline (SCD), a group known to be at increased risk for AD [28], found a positive association between the ‘high-Veggy’ dietary pattern, characterized by fruit, vegetables, fish, and fibers, with global cognition using a data-driven approach [30].

We aimed to cross-sectionally investigate the associations between dietary patterns and cognition in a memory clinic-based sample of German elderly free of dementia. More specifically, the association between adherence to the Mediterranean diet, MIND diet and data-derived dietary patterns and memory, language, executive functions, working memory and visuospatial functions was investigated.

## Methods

### Design

The DZNE-Longitudinal Cognitive Impairment and Dementia Study (DELCODE) is an observational longitudinal memory clinic-based multicenter (10 sites) study of the German Center for Neurodegenerative Diseases (DZNE) in Germany [31]. With the aim to study risk factors and early markers of Alzheimer’s disease, DELCODE is enrolling subjects with subjective cognitive decline (SCD), patients with mild cognitive impairment (MCI) or mild Alzheimer's dementia (AD), as well as first-degree relatives of patients with a documented diagnosis of AD and controls. All patient groups (SCD, MCI, AD) are referrals, including self-referrals. Controls and relatives of AD patients are recruited by standardized public advertisement. DELCODE participants receive extensive clinical and neuropsychological assessments, MRI and PET measurements, and biomaterials are collected. The study has been performed in accordance with the ethical standards laid down in the 1964 Declaration of Helsinki and its later amendments. Local ethical approval was obtained at each DELCODE site. All participants gave written informed consent. DELCODE is registered at the German Clinical Trials Register (DRKS00007966; 04/05/2015).

### Participants

#### Selection

From the DELCODE interim baseline data release (*N* = 687), all 395 participants free of dementia with dietary data available, were selected. Participants did not differ from non-participants regarding sex, education and MMSE score, but were slightly younger (69.3 ± 5.6 vs. 71.2 ± 5.7, *p* < 0.01). We excluded individuals with abnormal total energy intakes, i.e., below 2092 kJ (500 kcal) or above 20,920 kJ (5000 kcal) (*n* = 4) or with missing nutrition data over 20% (*n* = 2). This resulted in a study sample of 389 individuals (52% female, mean age 69 ± 6 years; mean Mini Mental State Examination score 29 ± 1).

#### Diagnostic groups

At study inclusion, the CERAD neuropsychological test battery was used to measure cognitive performance [31]. SCD was defined by the presence of subjectively reported decline in cognitive functioning with concerns as expressed to the physician of the memory center and a test performance of better than − 1.5 standard deviations (SD) below the age, sex, and education-adjusted normal performance on all subtests of the CERAD neuropsychological battery. Controls and AD-relatives were also required to have normal cognitive performance in all CERAD subtests. The MCI group consisted of individuals with demographically adjusted performance below − 1.5 SD on the delayed recall trial of the CERAD word-list episodic memory tests.

The current sample included 146 subjects with SCD (MMSE 29.2 ± 1.1), 60 patients with MCI (MMSE 28.0 ± 1.8), 35 first-degree relatives of AD dementia patients (MMSE 29.3 ± 1.1) and 148 healthy controls (no cognitive impairment; MMSE 29.5 ± 0.8).

## Measurements

### Cognition

At baseline, participants underwent an extensive neuropsychological assessment to capture cognitive functioning [32]. Cognitive data were transformed into five standardized cognitive domain scores: (1) memory (Alzheimer’s Disease Assessment Scale—Cognitive Subscale word list: trial 1, 2, 3, delayed recall and recognition; Free and Cued Selective Reminding Test (FCSRT): free recall and cue efficiency; Wechsler Memory Scale: logical memory 1 + 2; Consortium to Establish a Registry for Alzheimer's Disease (CERAD): figure savings, Symbol-Digit-Modalities Test (SDMT): incidental learning, Face Name Test); (2) language (verbal fluency: groceries and animals, Boston Naming Test (20 items); FCSRT: naming); (3) executive functioning (Trail Making Test A + B; Number Cancelation, SDMT, Flanker Task); (4) working memory (Digit Span Forward + Backward, FCSRT: interference task (Serial 3 s); and (5) visuospatial functioning (Clock copying + drawing, CERAD Figure copying).

#### Nutritional intake

Nutritional intake was assessed using the German version of the European Prospective Investigation of Cancer Food Frequency Questionnaire (EPIC-FFQ [33, 34]), a comprehensive 148-item, semi-quantitative self-report. The FFQ includes questions on type and amount of food items, frequency of consumption and methods for food preparation. Items were described either with household units or with usual portion sizes, visualized by illustrations. These data were used to calculate the nutritional intake in frequency per day/week and grams/day (calculations conducted by the German Institute of Nutritional Research (DIfE)). This resulted in 254 variables with information on either frequency per day/week or intake in grams/day.

### Dietary patterns

#### Mediterranean diet score

The original Mediterranean diet score is based on food groups (i.e., not food items) consumed by a traditional Greek population [35]. The diet score is a guideline to be used to rank people according to their adherence to the dietary guideline, not an exact diet. Numerous studies from diverse study populations across the globe have replicated the diet score based on the food items available on the assessment method in use [36]. The Mediterranean diet score included nine food groups (g/day): vegetables, legumes, fruits and nuts, dairy products, cereals, meat and poultry, fish, alcohol, and ratio of monounsaturated fatty acids and saturated fat [35]. Sex-specific medians based on the current study population were used as cut-offs. For healthy and unhealthy food groups, a score of 1 was given when intake was, respectively, equal or above the cut-off (e.g., vegetables) or below the cut-off (e.g., saturated fatty acids). Only for alcohol, a range indicating adherence was used. See Supplementary Table 2 for the cut-off per food group. Scores on all food groups were summed to calculate the total Mediterranean diet score, ranging from 0 to 9, with 9 indicating highest adherence to the Mediterranean diet guideline.

#### MIND diet score

The group of Professor M.C. Morris at Rush University Medical Center created the MIND diet by specifically including food groups that were shown to be related to brain health. These food groups were based on the published Mediterranean diet score and the DASH diet score, not taking the food items on the FFQ available in the Memory and Aging Project (MAP) into account. For example, MAP only had strawberries available to represent berries [15]. The MIND diet score comprises 15 food items, including 10 favorable items: green leafy vegetables, other vegetables, berries, nuts, whole grains, olive oil, beans, fish, poultry, and wine; and 5 non-favorable items: butter/margarine, cheese, red meat/ meat products, fast food/fried food, and pastry/sweets [16]. Population-specific foods were added to these categories (e.g., sauerkraut: fermented cabbage). Supplementary Table 3 presents the foods and cut-offs per food group. Olive oil consumption was scored 1 if used as the primary oil and 0 otherwise, according to the published MIND diet guideline. All other food groups were scored 0, 0.5 or 1 according to the frequency of consumption of each food item portion and/or when the intake of the food item adhered to the MIND diet score. Scores on all food groups were summed to calculate the total MIND score, ranging from 0 to 15, with 15 indicating highest adherence to the MIND diet guideline.

#### Data-derived dietary patterns

To derive data-driven dietary patterns, food variables were first grouped based on food groups (e.g., berries) as done previously with this FFQ [37], resulting in 39 food groups (see Supplementary Table 1 for details). Second, principle component analysis (PCA) was used to identify data-derived dietary patterns based on these 39 food groups. Assumptions for using this method were checked and met [38]. Specifically, we performed a PCA based on Spearman correlation (ranking of all individuals per food group). Factor loadings were calculated after Varimax rotation [39], to obtain components that are uncorrelated and, therefore, better interpretable. Factors were extracted based on Eigenvalues greater than 1.5 shown in the scree plot, based on experience that a lower cut-off would introduce uninterpretable a posteriori patterns.

#### Covariates

Data on height and weight were used to calculate BMI (kg/m^2^). Items of the PASE Questionnaire [40] were used as a proxy for physical activity, as we calculated the number of active days during the last week (e.g., walking, leisure activities, sports and household activities). Smoking status was divided into three categories: never smoked, former smoker and current smoker. Apolipoprotein E4 (APOE4)-status was included as a dichotomous variable (carrier vs non-carrier). To account for potential attrition bias, multiple imputation was used to create ten different copies of the original dataset and to replace missing values with imputed values. Imputed values were calculated from their predictive distribution based on the observed data [41]. Consequently, to account for uncertainty, results of the created datasets (*n* = 10) were combined and pooled in a pooled dataset (Supplementary table 4). Data that were imputed: education in years: *n* = 1 (0.3%); height: *n* = 2 (0.5%); weight: *n* = 4 (1.0%); physical activity score: *n* = 7 (1.8%) and smoking status: *n* = 2 (0.5%).

### Statistical analyses

Descriptive statistics were presented as mean (SD), median (interquartile range), or n (%) where appropriate. We used linear regression analyses to investigate the association between dietary patterns and cognition. First, analyses were adjusted for age, sex and education (Model 1). Then analyses were additionally adjusted for total daily energy intake (kcal), BMI, smoking status and physical activity and APOE4-status (Model 2). Model 2 was considered the fully adjusted model for the Mediterranean and MIND diet analyses. When analyzing the data-derived patterns, an additional Model 3 included all data-derived patterns simultaneously into the model in addition to Model 2 covariates. We used model 3 to assess the independent association of data-derived patterns with the outcomes and, therefore, considered Model 3 the fully adjusted model for these patterns. The fully adjusted models (Mediterranean diet + MIND: model 2, PCA: model 3) were also run after exclusion of MCI subjects to rule out that effects would be mainly driven, or rather obscured, by subjects with clinically significant cognitive impairment. Data are represented as unstandardized betas and 95% confidence intervals. A *p* value was considered significant when < 0.05. Analyses were conducted using SPSS version 22 [42].

## Results

Participants were on average 69.4 ± 5.6 years old, included 202 (52%) females, had a MMSE score of 29.1 ± 1.2 and had completed 14 (median; IQR 12–17) years of education. Descriptive statistics are listed in Table 1. The average Mediterranean diet score was 4.5 ± 1.9 (max. 9) and the average MIND diet score was 6.4 ± 1.4 (max. 15). MD and MIND scores were moderately correlated (*r* = 0.37) and did not differ between subgroups (SCD, REL, MCI, CON).

**Table 1:** Demographics

Demographic	*N* = 389
Age (years)	69.4 ± 5.6
Sex female *n* (%)	202 (51.9)
Education (years)^b^	14.0 (IQR: 12.0–17.0)
ApoE4-carrier *n* (%)^a^	107 (27.9)
BMI (kg/m^2^)	25.9 ± 3.7
Smoking status *n* (%)^b^	
Never smoked	192 (49.4)
Former smokers	165 (42.4)
Current smokers	32 (8.2)
Physical activity score^b^	358.4 ± 142.4
Total daily energy intake (kcal/day)	2315.4 ± 746.5
Cognitive status *n* (%)	
Cognitively normal	329 (85)
MCI	60 (15)
MMSE total score, max. 30	29.1 ± 1.2
Mediterranean diet, max. 9	4.5 ± 1.9
MIND diet, max. 15	6.4 ± 1.4

This table presents the demographics of the total study population. For continuous variables, mean and standard deviations are given, or median and interquartile range (IQR) if data were not normally distributed; for categorical variables, numbers and percentages are given. BMI: body mass index

*MCI* mild cognitive impairment, *MMSE* mini mental state examination

^a^APOEe4-status: *n* = 383, missing *n* = 6 (1.5%)

^b^Imputed data: education *n* = 1 (0.3%); physical activity score: *n* = 7 (1.8%); smoking status: *n* = 2 (0.5%)

First, associations of cognition with the Mediterranean and MIND diet scores were investigated (Table 2). Using model 1, we found associations between higher Mediterranean diet score and better memory (*p* = 0.003) and language (*p* = 0.017). In addition, higher MIND diet score was associated with better memory (*p* = 0.046). In the fully adjusted model, the associations of greater adherence to both the Mediterranean and MIND diet with better memory remained (*p* = 0.004 and *p* = 0.029, respectively), whereas the associations with language fell short of significance (Mediterranean *p* = 0.051, MIND *p* = 0.053). After the exclusion of subjects with MCI, associations of higher Mediterranean and MIND diet score with language became significant (Model 2: *p* = 0.031 and *p* = 0.027, respectively), while the association between MIND and memory was lost (*p* = 0.290).

**Table 2:** Mediterranean and MIND diet scores on cognitive outcomes

Domain	Group	Mediterranean diet	MIND diet
Memory			
Model 1	Total	0.051 [0.048 to 0.053]*	0.042 [0.001 to 0.082]*
Model 2	Total	0.049 [0.014 to 0.085]*	0.045 [0.003 to 0.087]*
Model 2	CN	0.037 [0.009 to 0.064]*	0.019 [− 0.014 to 0.051]
Language			
Model 1	Total	0.040 [0.023 to 0.057]*	0.036 [− 0.003 to 0.076]
Model 2	Total	0.033 [− 0.002 to 0.067]	0.039 [− 0.002 to 0.079]
Model 2	CN	0.030 [0.003 to 0.058]*	0.037 [0.004 to 0.069]*
Executive functioning			
Model 1	Total	0.018 [0.001 to 0.036]	0.013 [− 0.028 to 0.055]
Model 2	Total	0.013 [− 0.023 to 0.050]	0.014 [− 0.029 to 0.057]
Model 2	CN	0.014 [− 0.016 to 0.044]	0.014 [− 0.022 to 0.049]
Working memory			
Model 1	Total	0.025 [0.006 to 0.043]	.028 [− 0.014 to 0.070]
Model 2	Total	0.022 [− 0.017 to 0.060]	.031 [− 0.014 to 0.076]
Model 2	CN	0.025 [− 0.011 to 0.060]	.031 [− 0.011 to 0.073]
Visuospatial functioning			
Model 1	Total	0.002 [− 0.014 to 0.017]	.014 [− 0.021 to 0.049]
Model 2	Total	− 0.010 [− 0.042 to 0.022]	.014 [− 0.024 to 0.052]
Model 2	CN	− 0.006 [− 0.035 to 0.023]	.026 [− 0.008 to 0.059]

Presented are the unstandardized B and [95% CI’s]. Model 1: adjusted for age, sex and education (*n* = 389). Model 2: adjusted for age, sex, education, APOe4-status, total daily energy intake (kcal), BMI (kg/m^2^), smoking status and physical activity (*n* = 383). *p < 0.05. CN: cognitively normal (excluding MCI, *n* = 323)

Subsequently, with the use of PCA, we identified six data-derived dietary patterns, explaining 44% of the variance. We named the dietary patterns (1) ‘Warm meal’, (2) ‘Vegetables’, (3) ‘Cereals and nuts’, (4) ‘Alcoholic beverages’, (5) ‘Bread meal’ and 6) ‘Snacks’ based on the highest intake of the dietary pattern contents (Supplementary table 5: loading of food groups on PCA factors).

Groups did not differ regarding these patterns, except that subjects with MCI had lower scores for the ‘Alcoholic beverages’ component.

Table 3 presents the associations of the PCA-based dietary patterns with the cognitive domains. Higher ‘Alcoholic beverages’ dietary pattern score was associated with higher scores on all domains except for visuospatial functioning in all models. Specifically, we found that a higher ‘Alcoholic beverages’ dietary pattern score was related to better memory (*p* = 0.001), language (*p* = 0.001), executive functioning (*p* = 0.001) and working memory (*p* < 0.001). In addition, higher ‘Cereals and Nuts’ dietary pattern score was associated with better language (*p* = 0.041), but only in the fully adjusted model (Model 3). Using model 1, we found an association between higher adherence to the ‘Vegetables’ pattern and better memory (*p* = 0.039), which lost significance in models 2 (*p* = 0.059) and 3 (*p* = 0.080).

**Table 3:** Data-derived dietary patterns and cognitive outcomes

Domain	Model	Group	Warm meal	Vegetables	Cereals and nuts	Alcoholic beverages	Bread meal	Snacks
Memory	Model 1	Total	− 0.023 [− 0.081 to 0.036]	0.060 [0.003 to 0.118]*	0.024 [− 0.036 to 0.084]	0.095 [0.034 to 0.156]*	− 0.009 [− 0.067 to 0.048]	0.015 [− 0.043 to 0.074]
	Model 2	Total	− 0.029 [− 0.094 to 0.035]	0.058 [− 0.002 to 0.119]	00.035 [− 0.028 to 0.097]	0.093 [0.031 to 0.154]*	− 0.020 [− 0.084 to 0.045]	0.004 [− 0.062 to 0.071]
	Model 3	Total	0.021 [− 0.057 to 0.098]	0.057 [− 0.007 to 0.121]	0.061 [− 0.005 to 0.127]	0.108 [0.042 to 0.175]*	.000 [− 0.035 to 0.036]	0.046 [− 0.033 to 0.125]
	Model 3	CN	0.047 [− 0.013 to 0.107]	0.045 [− 0.005 to 0.096]	0.055 [.002 to 0.109]*	0.071 [0.018 to 0.124]*	.044 [− 0.009 to 0.098]	.051 [− 0.011 to 0.114]
Language	Model 1	Total	− 0.016 [− 0.072 to 0.040]	0.052 [− 0.003 to 0.108]	0.051 [− 0.007 to 0.109]	0.104 [0.046 to 0.162]*	.019 [− 0.037 to 0.074]	− 0.003 [− 0.060 to 0.053]
	Model 2	Total	− 0.027 [− 0.090 to 0.035]	0.040 [− 0.019 to 0.098]	0.046 [− 0.014 to 0.106]	0.099 [0.040 to 0.158]*	0.002 [− 0.060 to 0.064]	− 0.029 [− 0.093 to 0.035]
	Model 3	Total	0.010 [− 0.064 to 0.085]	0.035 [− 0.027 to 0.096]	00.067 [0.003 to 0.131]*	0.110 [0.046 to 0.174]*	.010 [− 0.057 to 0.077]	0.009 [− 0.068 to 0.085]
	Model 3	CN	− 0.005 [− 0.065 to 0.055]	.044 [− 0.006 to 0.094]	0.052 [− 0.001 to 0.105]	0.054 [0.001 to 0.107]*	.044 [− 0.009 to 0.097]	0.002 [− 0.060 to 0.065]
Exec. function	Model 1	Total	− 0.016 [− 0.075 to 0.043]	0.021 [− 0.037 to 0.080]	0.027 [− 0.034 to 0.088]	0.102 [0.041 to 0.163]*	− 0.001 [− 0.058 to 0.057]	− 0.007 [− 0.066 to 0.052]
	Model 2	Total	− 0.024 [− 0.090 to 0.043]	0.010 [− 0.052 to 0.072]	0.029 [− 0.035 to 0.093]	0.104 [0.042 to 0.167]*	− 0.016 [− 0.082 to 0.050]	− 0.022 [− 0.089 to 0.046]
	Model 3	Total	0.000 [− 0.079 to 0.079]	− 0.003 [− 0.068 to 0.063]	0.048 [− 0.020 to 0.116]	0.113 [0.045 to 0.181]*	− 0.020 [− 0.092 to 0.051]	− 0.001 [− 0.082 to 0.080]
	Model 3	CN	− 0.011 [− 0.077 to 0.001]	0.021 [− 0.077 to 0.056]	0.031 [− 0.028 to 0.090]	0.056 [− 0.002 to 0.115]	.027 [− 0.032 to 0.086]	0.009 [− 0.060 to 0.077]
Working mem	Model 1	Total	− 0.037 [− 0.099 to 0.025]	0.018 [− 0.043 to 0.079]	0.052 [− 0.012 to 0.116]	0.122 [0.058 to 0.185]*	− 0.010 [− 0.071 to 0.051]	− 0.007 [− 0.070 to 0.055]
	Model 2	Total	− 0.045 [− 0.114 to 0.025]	0.005 [− 0.060 to 0.070]	0.048 [− 0.019 to 0.115]	0.122 [0.056 to 0.187]*	− 0.023 [− 0.092 to 0.046]	− 0.019 [− 0.090 to 0.052]
	Model 3	Total	− 0.019 [− 0.100 to 0.063]	− 0.014 [− 0.083 to 0.054]	0.067 [− 0.004 to 0.138]	0.132 [0.061 to 0.202]*	− 0.034 [− 0.108 to 0.041]	− 0.003 [− 0.087 to 0.081]
	Model 3	CN	− 0.035 [− 0.112 to 0.043]	0.008 [− 0.057 to 0.073]	0.067 [− 0.001 to 0.136]	0.088 [0.019 to 0.156]*	.011 [− 0.058 to 0.080]	.009 [− 0.071 to 0.088]
Visuosp. Func	Model 1	Total	0.023 [− 0.029 to 0.075]	0.024 [− 0.027 to 0.075]	0.033 [− 0.021 to 0.087]	00.024 [− 0.031 to 0.078]	− 0.009 [− 0.060 to 0.042]	.004 [− 0.049 to 0.056]
	Model 2	Total	0.015 [− 0.043 to 0.073]	0.009 [− 0.045 to 0.063]	0.029 [− 0.027 to 0.085]	0.023 [− 0.032 to 0.079]	− 0.044 [− 0.102 to 0.013]	− 0.021 [− 0.080 to 0.038]
	Model 3	Total	0.012 [− 0.058 to 0.082]	− 0.003 [− 0.061 to 0.054]	0.032 [− 0.028 to 0.092]	0.031 [− 0.029 to 0.091]	− 0.046 [− 0.109 to 0.017]	− 0.015 [− 0.087 to 0.057]
	Model 3	CN	0.000 [− 0.062 to 0.062]	0.009 [− 0.043 to 0.062]	0.029 [− 0.026 to 0.84]	− 0.001 [− 0.056 to 0.054]	− 0.020 [− 0.076 to 0.035]	− 0.012 [− 0.015 to 0.010]

Presented are the results of the linear regression models between the PCA factors and cognitive outcomes. Reported are the unstandardized B and [95% CI’s]. Model 1: adjusted for age, sex and education (*n* = 389). Model 2: adjusted for age, sex, education, total daily energy intake (kcal), BMI (kg/m3), smoking status, physical activity and APOe4-status (*n* = 383). Model 3: model 2 with all PCA factors entered simultaneously. *p < 0.05. CN: cognitively normal, *N* = 323

After the exclusion of subjects with MCI, analyses were repeated using the fully adjusted model (Model 3). Higher adherence to the ‘Alcoholic beverages’ dietary pattern remained associated with better memory (*p* = 0.009), language (*p* = 0.045) and working memory (*p* = 0.012). The association with executive functions, however, attenuated and became non-significant (*p* = 0.060). Also, the association between the ‘Cereals and Nuts’ dietary pattern and better language attenuated in the cognitive unimpaired and became non-significant (*p* = 0.055). However, we did find an association between higher adherence to the ‘Cereals and Nuts’ dietary pattern and better memory (*p* = 0.042) in the cognitively normals.

## Discussion

In this study, we found that higher adherence to the Mediterranean diet or the MIND diet was associated with aspects of cognition in an elderly sample free of dementia, enriched with individuals at increased risk for AD. More specifically, higher adherence to MD and MIND was associated with better memory, and exclusion of MCI subjects revealed that Mediterranean and MIND diet were related to language in the unimpaired group. In addition, an empirical dietary pattern mainly characterized by intake of alcohol was associated with several cognitive domain scores. Although exclusion of MCI subjects attenuated the associations with this component, most remained significant.

These results are in line with population-based studies, which observed a positive association between adherence to the Mediterranean diet [43–46], or MIND diet and cognitive decline or dementia [15, 16, 18, 20]. While not all cross-sectional [21, 22] and longitudinal [47–49] studies did replicate these findings, a meta-analysis on the Mediterranean diet and a systematic review of the MIND diet support that greater adherence to these diets are positively associated with cognitive functioning, cognitive decline and AD [13, 50, 51]. Extending the evidence from these population-based studies, we here show that higher adherence to the Mediterranean diet and MIND diet are also associated with cognitive outcomes in a sample enriched with subjects who are either clinically or genetically at increased risk for AD.

The associations between the dietary patterns and cognition were most pronounced for memory and language. This has also been observed in some other studies [52, 53] and may be mediated by dietary effects on mediotemporal atrophy [53] or on cerebral amyloid pathology [54], which first affects memory function. Our language tasks assessed mainly verbal fluency performance. Verbal fluency was shown to be affected in individuals with amnestic MCI and cognitive complaints (e.g., [55]) and it relies on storage and retrieval of verbal information from semantic memory. As our sample was enriched for AD risk, and memory and language are affected early in the course of AD, it is tempting to speculate that the apparent specificity of the diet–cognition associations which we observed stems from protective effects of MEDI and MIND diets which mitigate early cognitive effects of AD. This hypothesis will need to be examined with biomarker data in DELCODE and other deeply phenotyped samples.

Regarding confounders, it is not likely that the associations that were found were driven by cardiovascular risk factors, because further adjustment for these diseases (data not shown) did not change the betas of the associations. After exclusion of individuals with cognitive impairment, some associations attenuated in significance or effect size. Analyses in larger samples are needed to investigate whether these changes indeed indicate different associations in different subgroups, or whether they were due to statistical power loss.

Longitudinal and imaging analyses in DELCODE will allow us to address sub-group analysis as well as the biological correlates of different dietary patterns.

A methodological issue with predefined dietary patterns is that they may not well represent the typical diet in many countries. We, therefore, supplemented the analysis with a data-driven approach. Two of the six data-derived dietary patterns also revealed a positive association with better cognition, PCA pattern 4 ‘Alcoholic beverages’ (being related to with better functioning in several domains), and PCA pattern 3 ‘Cereals and Nuts’ (being related to language in the total group and with memory in the cognitively normal group).

Our findings with respect to alcohol intake suggest a possibly beneficial effect of mild to moderate alcohol consumption for cognition, as has been described before in longitudinal studies [56–59]. Whereas light to moderate alcohol consumption might be beneficial for cognitive health, high alcohol intake or abuse of alcohol has been shown to be detrimental for brain function due to a U-shaped dose–response relationship [60, 61]. The majority of our sample had a moderate ethanol intake (total group mean 18.8 ± 24.4 g/day) which is in accordance with the alcohol guidelines of the Mediterranean diet (84% of females < 25 g/day, 88% of males < 50 g/day). Subjects with MCI had lower scores for PCA component 4, but the total amount of alcohol intake per day was not different, suggesting that all components of PCA4 contribute to the observed association and not solely the total alcohol intake. To rule out reversed causation, we excluded MCI patients and nearly all associations remained significant after exclusion. Thus, subjects worried about their memory for good reasons (i.e., subjects with MCI) may reduce their alcohol intake, but the observed associations between the “alcoholic beverages” component and cognition unlikely result from this change. It would be interesting for future studies to look into foods that often accompany alcohol intake, such as fish meals, that might also contribute to the observed positive association. Fruit was negatively loading on the ‘alcoholic beverages’ pattern. However, additional adjusting for fruit intake did not change the associations (data not shown).

Regarding the cereals and nuts, and bread meal pattern, we have found inconsistent results. Wholegrain and nuts are included in the Mediterranean diet and MIND guidelines, because their potential beneficial health effects can primarily be ascribed to fiber and phytonutrients [62, 63]. However, the inconsistent findings of the present study are in line with previous studies that did not find a relationship between wholegrain and cognitive function or decline [64, 65]. Results from studies that investigated nuts in relation to both outcomes have been mixed, as some found associations, while others did not [48, 66–68].

The primary strength of this study involved the study population, as the study was conducted in a sample of elderly that were free of dementia, enriched with individuals at increased risk for AD. Results from the present study are a significant addition to the current literature, which is mainly based on population-based samples. Second, we used an extensive neuropsychological test battery to assess cognitive functioning, which is more sensitive to differences compared to measures of global cognitive functioning such as the MMSE. Third, we used a state-of-the-art psychometrics method to aggregate neuropsychological scores into cognitive domains scores. Fourth, we used a detailed food frequency questionnaire, providing information on the amount and frequency of nutrient and food intake, which was modified specifically for a German population, which enabled us to use a combination of both predefined and data-derived dietary patterns to assess adherence to dietary patterns in this study population.

This study has several limitations. The cross-sectional study design precludes conclusions about temporality of associations or causality. Longitudinal data will become available in DELCODE and will be used to study whether diet is associated with future cognitive decline. The analyzed sample consists of several subgroups, which we analyzed together as they did not differ regarding diet and—with the exception of MCI patients—performed within normal limits at neuropsychological testing. Individuals with amnestic MCI might have had difficulties in filling out the FFQ. However, all study participants had a study partner (e.g., spouse) to assist and we excluded questionnaires indicating an unrealistic nutritional intake. It has also been reported that FFQs from MCI patients are valid if a study partner is involved [69]. We used a FFQ, which is subject to measurement error. To account for potential systematic measurement error, we adjusted the relationships under study for total energy intake [70]. However, we acknowledge the possible presence of non-differential misclassification, which may have led to bias towards the null [71]. Furthermore, while the subjects with SCD and with an AD relative may be at increased risk for having preclinical AD, they are generally healthy elderly research participants similar to those in the volunteer control group. Subgroup analyses (e.g., in subjects with Amyloid pathology) are planned once the complete sample and biomarker data will be available. All participants were German and mainly well educated, white individuals, limiting the generalizability of the findings to other countries or cultures. While our sample is heterogeneous in terms of recruitment, it represents a group of elderly which is enriched for AD-risk, but free of dementia. Importantly, our results align well with data from general population studies, suggesting that the association of diet with specific cognitive functions also holds true in risk-enriched populations, which is a prerequisite for considering nutritional intervention studies in such groups. As associations between diet and cognition are generally small, and the size of our sample was only moderate, we did not correct for multiple testing. While this limitation calls for a cautious interpretation, we note that the size and pattern of the associations found are consistent with much of the literature. Replication of our findings in other risk-enriched cohorts, the study of imaging and fluid neurodegeneration biomarkers in relation to diet, and longitudinal studies are needed to corroborate the present results. The current results add to current literature on diet and cognition as a sample including individuals with increased risk for AD was studied and an elaborate cognitive assessment has been used to construct sensitive cognitive outcome measures.

In this sample of German elderly free of dementia, Mediterranean diet and MIND diet were related to better memory and language, and two data-derived dietary patterns, one characterized by high intake of cereals and nuts and the other one by higher intake of alcoholic beverages were also related to better cognition.

The present study results would be consistent with a beneficial effect of some diets and dietary components on cognitive health. We consider it encouraging that associations previously found in population samples were also evident in our risk-enriched sample, which can be conceived as a target sample for prevention of cognitive decline, as subjects often are concerned about their cognitive health and motivated to change their lifestyle. It may not be “too late” for dietary changes to be effective in such populations.

In sum, the current results suggest that dietary intake is of importance in individuals free of dementia, ranging from cognitively healthy to MCI, and should be taken into account when designing interventions to delay cognitive decline.

## Electronic supplementary material

Below is the link to the electronic supplementary material.

Supplementary file1 (DOCX 30 kb)

## Abbreviations

AD: Alzheimer’s disease
IQR: Interquartile range
MCI: Mild cognitive impairment
MIND: Mediterranean-DASH Intervention for Neurodegenerative Delay
PCA: Principal component analysis
