# Supplementary material for: Dietary patterns are related to cognitive functioning in elderly enriched with individuals at increased risk for Alzheimer’s disease
Source: Eur J Nutr. 2020 May 29;60(2):849–60. doi: 10.1007/s00394-020-02257-6 (PMC7900077; doi:10.1007/s00394-020-02257-6)
Supplement: Supplementary file 1 — Supplementary file1 (DOCX 30 kb) [file 394_2020_2257_MOESM1_ESM.docx]

**Supplement 1: Mediterranean diet score cut-offs.**

Mediterranean diet scoring[35], with population-specific median cut-off per sex.

| Food group | Gender | Intake [g/d] | Mediterranean diet score |
| --- | --- | --- | --- |
| Vegetables | *Men*  *Women* | *< 583.6*  *≥ 583.6*  *< 536.1*  *≥ 536.1* | *0*  *1*  *0*  *1* |
| Legumes | *Men*  *Women* | *< 10.4*  *≥ 10.4*  *< 7.9*  *≥ 7.9* | *0*  *1*  *0*  *1* |
| Fruits and nuts | *Men*  *Women* | *< 393.0*  *≥ 393.0*  *< 385.7*  *≥ 385.7* | *0*  *1*  *0*  *1* |
| Dairy products | *Men*  *Women* | *≥ 222.6*  *< 222.6*  *≥ 216.2*  *< 216.2* | *0*  *1*  *0*  *1* |
| Cereals | *Men*  *Women* | *< 191.0*  *≥ 191.0*  *< 145.7*  *≥ 145.7* | *0*  *1*  *0*  *1* |
| Meat and poultry | *Men*  *Women* | *≥ 129.3*  *< 129.3*  *≥ 94.9*  *< 94.9* | *0*  *1*  *0*  *1* |
| Fish | *Men*  *Women* | *< 26.4*  *≥ 26.4*  *< 21.7*  *≥ 21.7* | *0*  *1*  *0*  *1* |
| Alcohol | *Men*  *Women* | *< 10 and > 50*  *10-50*  *< 5 and > 25*  *5-25* | *0*  *1*  *0*  *1* |
| Ratio MUFA/SFA | *Men*  *Women* | *< 1.8*  *≥ 1.8*  *< 1.8*  *≥ 1.8* | *0*  *1*  *0*  *1* |
| Total Mediterranean diet score |  | | *0-9* |

G: grams; d: day; MUFA: monounsaturated fatty acids; SFA: saturated fatty acids

**Supplement 2a: MIND diet score cut offs**

MIND diet scoring [15,16]

| **MIND diet component** | **Servings** | **MIND score** |
| --- | --- | --- |
| Green leafy vegetables | ≥ 6 / wk  > 2 to < 6 / wk  ≤ 2 / wk | 1  0.5  0 |
| Other vegetables | ≥ 1 / d  5 to < 7 / wk  < 5 / wk | 1  0.5  0 |
| Berries | ≥ 2 / wk  1 / wk  < 1 / wk | 1  0.5  0 |
| Nuts | ≥ 5 / wk  1 / mo to < 5 / wk  < 1 / mo | 1  0.5  0 |
| Olive oil | Primary oil used  Not primary | 1  0.5  0 |
| Butter, Margarine | < 1 T / d  1 to 2 / d  > 2 T / d | 1  0.5  0 |
| Cheese | < 1 / wk  1 to 6 / wk  7+ / wk | 1  0.5  0 |
| Whole Grains | ≥ 3 / d  1 to 2 / d  < 1 / d | 1  0.5  0 |
| Fish (not fried) | ≥ 1 meals / wk  1 to 3 / mo  Rarely | 1  0.5  0 |
| Beans | > 3 meals /wk  1 to 3 / wk  < 1 meal / wk | 1  0.5  0 |
| Poultry (not fried) | ≥ 2 meals / wk  1 / wk  < 1 meal / wk | 1  0.5  0 |
| Red Meat and products | < 4 meals / wk  4 to 6 / wk  7+ meals / wk | 1  0.5  0 |
| Fast Fried Food | < 1 time / wk  1 to 3 / wk  4+ times / wk | 1  0.5  0 |
| Pastries & Sweets | < 5 / wk  5 to 6 / wk  7+ / wk | 1  0.5  0 |
| Wine | 1 glass / d  1 / mo to 6 / wk  > 1 glass / d or never | 1  0.5  0 |
| **Total MIND Score** |  | **0-15** |

**Supplement 2b: Foods per food group for the MIND diet score**

| **MIND diet food groups** | **Morris et al. (2015) [15,16]** | **DELCODE** |
| --- | --- | --- |
| **Green leafy vegetables** | kale, collards, greens; spinach; lettuce/tossed salad | green salads, leaf salad mix, spinach |
| **Other vegetables** | green/red peppers, squash, cooked carrots, raw carrots, broccoli, celery, potatoes, peas or lima beans, potatoes*, tomatoes, tomato sauce, string beans, beets, corn, zucchini/summer, squash/eggplant, coleslaw, potato salad* | eggplant, zucchini, avocado, broccoli,  cauliflower, kohlrabi, vegetable mix,  carrot raw/cooked, mushrooms,  cucumber, gherkin, sauerkraut,  asparagus, tomato raw/cooked, bell pepper raw/cooked, tomato sauce, white cabbage,  green cabbage, brussels sprouts, red cabbage, olives, onions, garlic |
| **Berries** | Strawberries | Berries (e.g. strawberries, gooseberries, blueberries) |
| **Nuts** | Nuts | Nuts – fresh, roasted, salted |
| **Olive oil** | Olive oil | Olive oil |
| **Butter, Margarine** | Butter, margarine | butter, margarine, half-fat margarine, lard |
| **Cheese** | Cheese | Cream cheese, grainy cream cheese,  Sour milk cheese, semi-hard cheese,  hard cheese, soft cheese |
| **Whole Grains** | Whole Grains | Whole Grains (bread, bread rolls, toast) |
| **Fish (not fried)** | tuna sandwich, fresh fish as main dish; not fried fish cakes, sticks, or sandwiches | fish (fresh, cans) |
| **Beans** | Beans, lentils, soybeans | Legumes |
| **Poultry (not fried)** | chicken or turkey sandwich, chicken or turkey as main dish and never eat fried at home or away from home | Poultry |
| **Red meat and products** | cheeseburger, hamburger, beef tacos/burritos, hot dogs/sausages, roast beef or ham sandwich, salami, bologna, or other deli meat sandwich, beef (steak, roast) or lamb as main dish, pork or ham as main dish, meatballs or meatloaf | pork sausage, Bierwurst, Bratwurst, ham sausage, meatball, Boulette, poultry sausage, veal, lamb, rabbit,  game, liver sausage,  beef, raw ham,  boiled ham, black pudding,  brawn, salami, pork/beef sausage, cabanossi, pork |
| **Fast Fried Foods** | Fried food away from home (like French fries, chicken nuggets) | potato preparations, fish preparations |
| **Pastries & Sweets** | biscuit/roll, poptarts, cake, snack cakes/twinkies, Danish/sweetrolls/pastry, donuts, cookies, brownies, pie, candy bars, other candy, ice cream, pudding, milkshakes/frappes | desserts, egg pancakes,  waffles, yeast pastries, yeast cakes,  cheesecake, cream cake, cookies, biscuits,  sponge cake, sweets  without chocolate, chocolate,  sweets with chocolate, |
| **Wine** | Wine | red wine, white wine, rosé wine |

*in the original paper from Morris et al. potatoes and potato salad are listed; however, these have been mistakenly listed upon request by Morris et al. (2015)

**Supplement 3: Food grouping**

Foods (grams/day) were grouped as described below:

| **Food group** | **Foods** |
| --- | --- |
| Potatoes | Potatoes |
| Leafy vegetables | Leafy vegetables |
| Fruiting and root vegetables | Fruiting vegetables, root vegetables |
| Cabbages | Cabbages |
| Other vegetables | Mushrooms, grain pod vegetables, onion, Garlic, stalk vegetables, sprouts, mixed salad, mixed vegetables |
| Legumes | Legumes |
| Fruits | Fruits |
| Nuts | Nuts, seeds |
| Other fruits | Mixed fruits, olives |
| Milk, dairy products | Milk, milk beverages, yogurt |
| Cheese | Fromage blanc, petites suisses, cheeses |
| Desserts | Cream desserts, puddings, dairy creams, ice cream, ice cream/water ice unclassified |
| Pasta rice | Pasta, rice, other grain |
| Bread | Bread, crispbread rusks |
| Breakfast cereals | Breakfast cereals |
| Other cereals | Salty biscuits, aperitif biscuits, dough, pastry, flour flakes, starches, semolina |
| Red meat | Beef, veal, pork, mutton, lamb, rabbit. |
| Poultry | Other poultry, chicken |
| Processed meat | Processed meat |
| Fish | Fish, Crustaceans molluscs, fish products, fish crumbs |
| Eggs | Egg |
| Margarine | Margarines |
| Vegetable oils | Vegetable oils |
| Butter | Butter |
| Sugar Confectionary | Sugar honey jam, chocolate candy bars paste, confectionery non chocolate, syrup |
| Cakes cookies | Cakes, pies, pastries, puddings, dry cakes, biscuits |
| Fruit and vegetable juices | Fruit and vegetable juices |
| Soft drinks | Carbonated soft isotonic drinks |
| Tea | Tea, Herbal tea |
| Coffee | Coffee |
| Water | Water |
| Wine | Wine |
| Beer | Beer, cider |
| Spirits | Spirits, brandy |
| Other Alcoholic beverages | Aniseed drinks, liquors, cocktails punches |
| Sauces | Sauces unclassified, tomato sauces, dressing sauces, mayonnaises, dessert sauces. |
| Condiments | Spices, herbs, flavorings, condiments |
| Soups | Soups, bouillon |
| Snacks | Snacks |

**Supplement 4: Specification of the multiple imputation procedure**

| Software | IBM SPSS Statistics for Windows (Release 23.0) |
| --- | --- |
| Imputation method | Fully conditional specification (Markov chain Monte Carlo method) |
| Key settings | Maximum iterations: 20 |
| Imputed data sets | 10 |
| Variables included in the | Diagnosis; Mediterranean diet single scores; MIND diet single scores; |
| imputation procedure | verbal memory; language; executive function; working memory; |
| (imputed or used as | visuospatial functions; global cognitive score; age (y); gender; |
| predictors of missing) | education; APOE4 status; BMI (kg/m²); physical activity; energy intake (kcal); smoking; diabetes; hypertension; hyperlipidemia; hypercholesterolemia; depression; stroke |
|  |  |
| Additionally added | height (cm); weight (kg); cigarettes per day |
| predictive variables to |  |
| increase plausibility of |  |
| missing at random |  |

Abbreviations: MIND= Mediterranean-DASH Intervention for Neurodegenerative Delay; cm= centimetres; kg= kilogram; y= years.

**Supplement 5: loading of food groups on data-derived dietary patterns**

|  | 1  ‘Warm meal’ | 2  ‘Vegetables’ | 3  ‘Cereals and nuts’ | 4  ‘Alcoholic beverages’ | 5  ‘Bread meal’ | 6  ‘Snacks’ |
| --- | --- | --- | --- | --- | --- | --- |
| Red meat | ,696 |  |  |  |  |  |
| Processed meat | ,653 |  |  |  |  |  |
| Legumes | ,606 |  |  |  |  |  |
| Potatoes | ,584 |  |  |  |  |  |
| Margarine | ,537 |  |  |  |  |  |
| Sauces | ,510 |  |  |  |  |  |
| Poultry | ,477 |  |  |  |  |  |
| Soft drinks | ,378 | -,337 |  |  |  |  |
| Soups | ,360 |  | ,344 |  |  |  |
| Eggs | ,307 |  |  |  |  |  |
| Vegetable oils |  | ,803 |  |  |  |  |
| Fruiting & rooting vegetables |  | ,745 |  |  |  |  |
| Leafy vegetables |  | ,717 |  |  |  |  |
| Other vegetables | ,555 | ,609 |  |  |  |  |
| Cabbages | ,430 | ,595 |  |  |  |  |
| Condiments |  | ,459 |  |  |  |  |
| Tea |  | ,328 |  |  |  |  |
| Breakfast cereals |  |  | ,728 |  |  |  |
| Other cereals |  |  | ,697 | ,309 |  |  |
| Nuts |  |  | ,639 |  |  |  |
| Snacks |  |  | ,537 | ,336 |  |  |
| Milk & dairy products |  |  | ,449 |  |  |  |
| Fruit & vegetable juices |  |  | ,362 |  |  |  |
| Pasta & rice |  |  | ,341 | ,301 |  |  |
| Beer |  |  |  | ,714 |  |  |
| Spirits |  |  |  | ,700 |  |  |
| Wine | -,363 |  |  | ,522 |  |  |
| Fruits |  | ,425 |  | -,483 |  | ,321 |
| Fish |  |  |  |  |  |  |
| Butter |  |  |  |  | ,720 |  |
| Bread |  |  |  |  | ,693 |  |
| Sugar confectionary |  |  |  |  | ,625 |  |
| Other alcoholic beverages |  |  |  | ,371 |  | ,585 |
| Desserts |  |  |  |  |  | ,556 |
| Cheese |  |  |  |  |  | ,477 |
| Cakes & cookies | ,308 |  |  |  | ,369 | ,449 |
| Water |  |  |  |  |  | ,326 |
| Coffee |  |  |  |  |  | ,301 |
| Other Fruits |  |  |  |  |  |  |

*NOTE: This Table present the loading of the food groups on the PCA-derived dietary components. Loadings <.3 are not included to increase readability.*
